# Pulmonary Vein Isolation Alone Versus Adjunctive Ablation for Paroxysmal Atrial Fibrillation

**DOI:** 10.1111/jce.70519

**Published:** 2026-10-06

**Authors:** Michel Abou Khalil, Christian M. Massad, Eli Tsakiris, William Herbst, Maximilian Moersdorf, Mohammad Montaser Atasi, Yara Menassa, Alex El Darzi, Carlo El Khoury, Ghassan Bidaoui, Hadi Younes, Yishi Jia, Rodolfo Montiel, Amitabh Pandey, Han Feng, Qussay Marashly, Nassir F. Marrouche, Omar Kreidieh

**Affiliations:** ^1^ Tulane Research Innovation for Arrhythmia Discovery, Tulane University School of Medicine New Orleans Louisiana USA

**Keywords:** antiarrhythmic drugs, arrhythmia recurrence, atrial fibrillation, atrial flutter, catheter ablation, pulmonary vein isolation, real‐world evidence

## Abstract

**Background:**

Pulmonary vein isolation (PVI) is the standard ablation strategy for paroxysmal atrial fibrillation (PAF). Despite guideline recommendations favoring PVI alone in PAF, some operators routinely perform extra pulmonary vein ablation (PVI+) during initial procedures.

**Objective:**

To determine frequency of PVI+ strategy for treatment of PAF in the real world and examine its impact on arrhythmic recurrence and progression compared to PVI alone.

**Methods:**

Using the TriNetX research network, we identified adults with PAF who underwent catheter ablation between January 2020 and January 2024. Recurrence was defined as repeat ablation, cardioversion, or antiarrhythmic drug (AAD) use beyond a 3‐month blanking period. Progression to persistent AF and atypical atrial flutter were also assessed. Kaplan‐Meier analysis was performed with 3‐year follow‐up.

**Results:**

Before matching, 5134 of 18,891 patients (27.2%) underwent PVI+. In the propensity matched analysis (*n* = 5020 per group), recurrence was significantly higher in the PVI+ group (HR 1.28, 95% CI 1.16–1.42; *p* < 0.001). Repeat ablation, cardioversion and AAD use were more common with PVI+ (HR 1.99, 95% CI 1.60–2.46; *p* < 0.0001, HR 1.37, 95% CI 1.13–1.66; *p* = 0.001, and HR 1.41, 95% CI 1.05–1.87; *p* = 0.020, respectively). Progression to persistent AF was higher with PVI+ (HR 1.37, 95% CI 1.23–1.53, *p* < 0.001), as was atypical atrial flutter (HR 2.63, 95% CI 1.97–3.50; *p* < 0.0001).

**Conclusion:**

Extra‐pulmonary vein ablation during first‐time PAF ablation remains common in real‐world practice but is associated with worse outcomes including repeat ablation, cardioversion, AAD use, atrial flutter, and progression to persistent AF.

## Graphical abstract

Clinical Outcomes of Pulmonary Vein Isolation Alone Versus Additional Lesion Sets in Paroxysmal Atrial Fibrillation

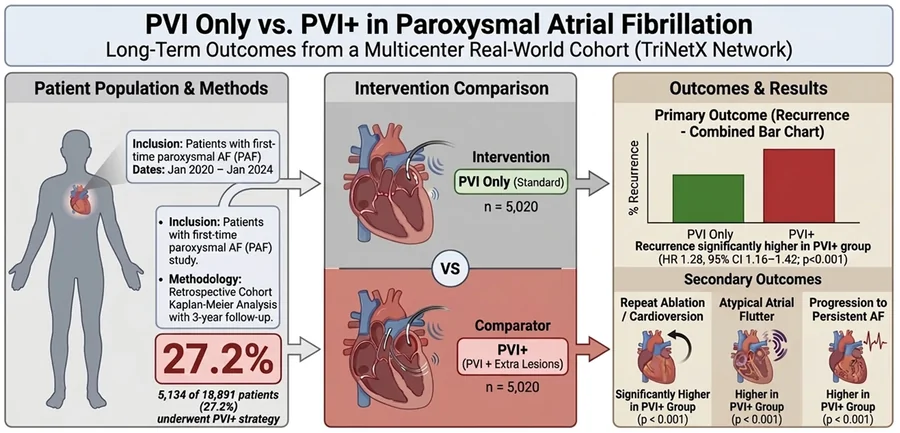

**Abbreviations**
AAD: antiarrhythmic drug
AF: atrial fibrillation
CI: confidence interval
CPT: current procedural terminology
HR: hazard ratio
ICD‐10: International Classification of Diseases, 10th Revision
LVA: low‐voltage area
PAF: paroxysmal atrial fibrillation
PFA: pulsed field ablation
PSM: propensity score matching
PVI: pulmonary vein isolation
PVI+: pulmonary vein isolation with additional ablation
SD: standard deviation
TIA: transient ischemic attack
WACA: wide‐area circumferential ablation

## 1 Introduction

Catheter ablation has been shown to improve hard clinical outcomes, including mortality, stroke, and heart failure hospitalization, across diverse populations [1, 2]. For patients with paroxysmal atrial fibrillation (PAF), it is an established rhythm‐control strategy, with pulmonary vein isolation (PVI) serving as the cornerstone of ablation therapy. Pulmonary veins are the dominant trigger for AF initiation, and durable electrical isolation of the pulmonary veins has been shown to significantly reduce arrhythmia recurrence compared with medical therapy alone [3]. Consequently, contemporary professional society guidelines recommend PVI as the primary ablation strategy for patients with PAF undergoing first‐time ablation [4].

Despite these recommendations, substantial variation exists in real‐world ablation practice. Many operators routinely perform additional extra‐pulmonary vein ablation (PVI+) during index procedures including linear or focal lesions, posterior wall isolation, complex fractionated atrial electrogram and empiric substrate modification [5]. This practice seems to contradict the results of most randomized trials in PAF where extrapulmonary vein ablation has generally failed to demonstrate benefit [4].

Given the above uncertainties we sought to evaluate the frequency of PVI+ strategy and its impact on AF related outcomes, progression to persistent AF, and post‐ablation atrial flutter in routine clinical practice. For these purposes, we collated data using the TriNetX research network, a multi‐institutional database aggregating real‐world electronic medical records.

## 2 Methods

### 2.1 Data Source and Study Design

We conducted a retrospective observational cohort study using data from the TriNetX Research Network, a global federated health research platform that provides access to de‐identified electronic health records (EHRs) from 109 participating healthcare organizations (HCOs). The TriNetX database includes longitudinal data on patient demographics, diagnoses, procedures, medications, laboratory values, and mortality. All data are aggregated and de‐identified in accordance with the Health Insurance Portability and Accountability Act (HIPAA) de‐identification standards (Section 164.514[a]). As investigators do not have access to identifiable patient information, this study was exempt from institutional review board approval and informed consent requirements.

### 2.2 Cohort Definitions

Adult patients (≥ 18 years) with a diagnosis of paroxysmal atrial fibrillation (PAF) who underwent an index catheter ablation procedure between January 1, 2020, and January 5, 2024, were identified using ICD‐10 and CPT codes. Patients with any diagnosis of persistent atrial fibrillation, longstanding persistent atrial fibrillation, permanent atrial fibrillation, or typical or atypical atrial flutter prior to the index procedure were excluded.

Two mutually exclusive cohorts were defined:

- PVI+ cohort (Cohort 1): Patients who underwent pulmonary vein isolation (PVI; CPT 93656) with additional linear or focal left or right atrial ablation during the same procedure, defined by CPT code 93657.
- PVI‐only cohort (Cohort 2): Patients who underwent PVI alone (CPT 93656) without additional atrial ablation of atrial fibrillation (absence of CPT 93657).

Only patients in whom the qualifying ablation procedure occurred within 2 years of the documented PAF diagnosis were included. The codebook used to define our patient cohorts and outcomes is provided in Table 1.

**Table 1:** Codebook for exposure and outcome definitions.

Term	Meaning	Coding system	Code(s)
Exposure–PVI only	Pulmonary vein isolation	CPT	93 656
Exposure–PVI+	Adjunctive linear/focal atrial ablation	CPT	93 657 + 93 656
Inclusion diagnosis	Paroxysmal atrial fibrillation	ICD‐10‐CM	I48.0
Exclusion AF diagnoses	Persistent AF forms	ICD‐10‐CM	I48.3, I48.4, I48.11, I48.19, I48.1, I48.92
Antiarrhythmics	Amiodarone	RxNorm	703
	Dronedarone	RxNorm	2 33 698
	Sotalol	RxNorm	9947
	Propafenone	RxNorm	8754
	Quinidine	RxNorm	9068
	Dofetilide	RxNorm	49247
	Flecainide	RxNorm	4441
Recurrence–procedures	Repeat PVI/redo ablation	CPT	93 656, 93 657
	Electrical cardioversion	CPT	92 960, 92 961
Recurrence–ICD‐10‐PCS	Rhythm restoration	ICD‐10‐PCS	5A2204Z
AF progression	Persistent AF forms	ICD‐10‐CM	I48.1, I48.19, I48.11
Atypical flutter	Atypical atrial flutter	ICD‐10‐CM	I48.4
Mortality	Death	Demographic flag	N/A
Stroke/TIA	Cerebral infarction/TIA	ICD‐10‐CM	I63, G45
HF hospitalization	Acute HF variants	ICD‐10‐CM	I50.21, I50.31, I50.811, etc.
Inpatient stay	Hospital encounter	CPT	10 13 659

### 2.3 Index Event and Follow‐up

For both cohorts, the index event was defined as the date of the qualifying catheter ablation procedure (PVI+ or PVI‐only). Outcomes were assessed within a predefined time window starting 90 days after the index event to account for the post‐ablation blanking period and extending to 1095 days (3 years) following the index event. Patients were censored at the time of last recorded clinical encounter or death.

### 2.4 Study Outcomes

The primary outcome was clinically significant atrial arrhythmia recurrence, defined as the occurrence of any repeat catheter ablation, electrical cardioversion, or usage of class I or III antiarrhythmic drug therapy (including amiodarone, sotalol, propafenone, quinidine, procainamide, dofetilide, or dronedarone) following the 90‐day blanking period. Secondary outcomes included repeat ablation procedures, electrical cardioversion, progression to persistent atrial fibrillation, and development of atypical atrial flutter. All outcomes were defined using validated ICD‐10, CPT, ICD‐10‐PCS, and RxNorm codes as specified within the TriNetX outcomes framework.

### 2.5 Statistical Analysis

Baseline characteristics were compared between cohorts using descriptive statistics. Continuous variables are reported as mean ± standard deviation and categorical variables as counts and percentages.

To minimize confounding, propensity score matching (PSM) was performed using a 1:1 nearest‐neighbor matching algorithm without replacement. Covariates included demographics (age, sex, race, ethnicity), cardiovascular comorbidities (hypertension, diabetes mellitus, ischemic heart disease, cardiomyopathy, heart failure subtypes, prior stroke or transient ischemic attack, chronic kidney disease, obstructive sleep apnea), medication use (beta‐blockers, ACE inhibitors, ARBs, diuretics, mineralocorticoid receptor antagonists, anticoagulants, and antiarrhythmic drugs), and body mass index. Balance between cohorts was assessed using standardized mean differences, with values < 0.1 indicating adequate balance.

To assess whether AAD use was driving the primary outcome, a sensitivity analysis was performed with recurrence redefined as repeat ablation or cardioversion only, excluding AAD use.

Time‐to‐event analyses were performed using Kaplan–Meier survival curves, with between‐group comparisons assessed using the log‐rank test. Cox proportional hazards models were used to estimate hazard ratios (HRs) with 95% confidence intervals (CIs). All analyses were conducted within the TriNetX Analytics environment, which utilizes R (version 4.0.2). A two‐sided *p*‐value < 0.05 was considered statistically significant.

## 3 Results

### 3.1 Study Population

We identified a total of 18,891 patients with paroxysmal atrial fibrillation who met our inclusion criteria and underwent catheter ablation between January 1, 2020, and January 5, 2024 at 109 healthcare organizations participating in the TriNetX Research Network. Of those patients, 27.2% (5134) underwent a PVI+ strategy while 72.8% underwent a PVI‐only strategy. After 1:1 propensity score matching for demographic, clinical, medication, and laboratory variables, 5020 patients remained in each cohort with well‐balanced baseline characteristics (standardized mean differences < 0.1 across all covariates) Table 2. Baseline characteristics before matching, with standardized mean differences, are provided in Supporting Information S1: Table S1. The mean age after matching was 67.1 ± 10.5 years in the PVI+ group and 67.1 ± 10.2 years in the PVI‐only group, with 43.7% and 43.8% female patients, respectively.

**Table 2:** Baseline characteristics after propensity score matching.

Variable	PVI+ (*n* = 5020)	PVI‐Only (*n* = 5020)	*p*‐value
Age, mean ± SD	67.1 ± 10.5	67.1 ± 10.2	0.880
Female sex, %	43.7%	43.8%	0.920
White race, %	87.5%	87.2%	0.631
Black race, %	5.2%	5.1%	0.822
Unknown ethnicity, %	10.0%	10.2%	0.765
Hypertension, %	74.5%	73.6%	0.285
Diabetes, %	22.3%	21.6%	0.426
COPD, %	9.8%	9.7%	0.788
Heart failure, %	26.1%	26.3%	0.785
Ischemic heart disease, %	44.1%	43.7%	0.702
TIA/stroke, %	7.1%	6.8%	0.504
CKD stage ≥ 3, %	10.6%	10.2%	0.601
OSA, %	33.6%	33.2%	0.688
BMI, mean ± SD	30.4 ± 6.4	30.6 ± 6.4	0.329
Antiarrhythmic use, %	79.7%	79.0%	0.336
Anticoagulation, %	89.2%	89.2%	1.000

### 3.2 Primary Outcome: Atrial Arrhythmia Recurrence

Clinically significant atrial arrhythmia recurrence occurred in 777 patients (15.5%) in the PVI+ group and 710 patients (14.1%) in the PVI‐only group. On Kaplan–Meier analysis, the PVI+ group demonstrated a significantly higher cumulative incidence of recurrence compared with the PVI‐only group (log‐rank *p* < 0.001) (Figure 1). In Cox proportional hazards analysis, PVI+ was associated with a 28% higher risk of recurrence (HR 1.28; 95% CI 1.16–1.42). In a sensitivity analysis excluding AAD use from the recurrence definition, PVI+ remained associated with a higher risk of recurrence (HR 1.447; 95% CI 1.271–1.647; *p* < 0.001), consistent with the primary analysis (Figure 1).

**Figure 1:**
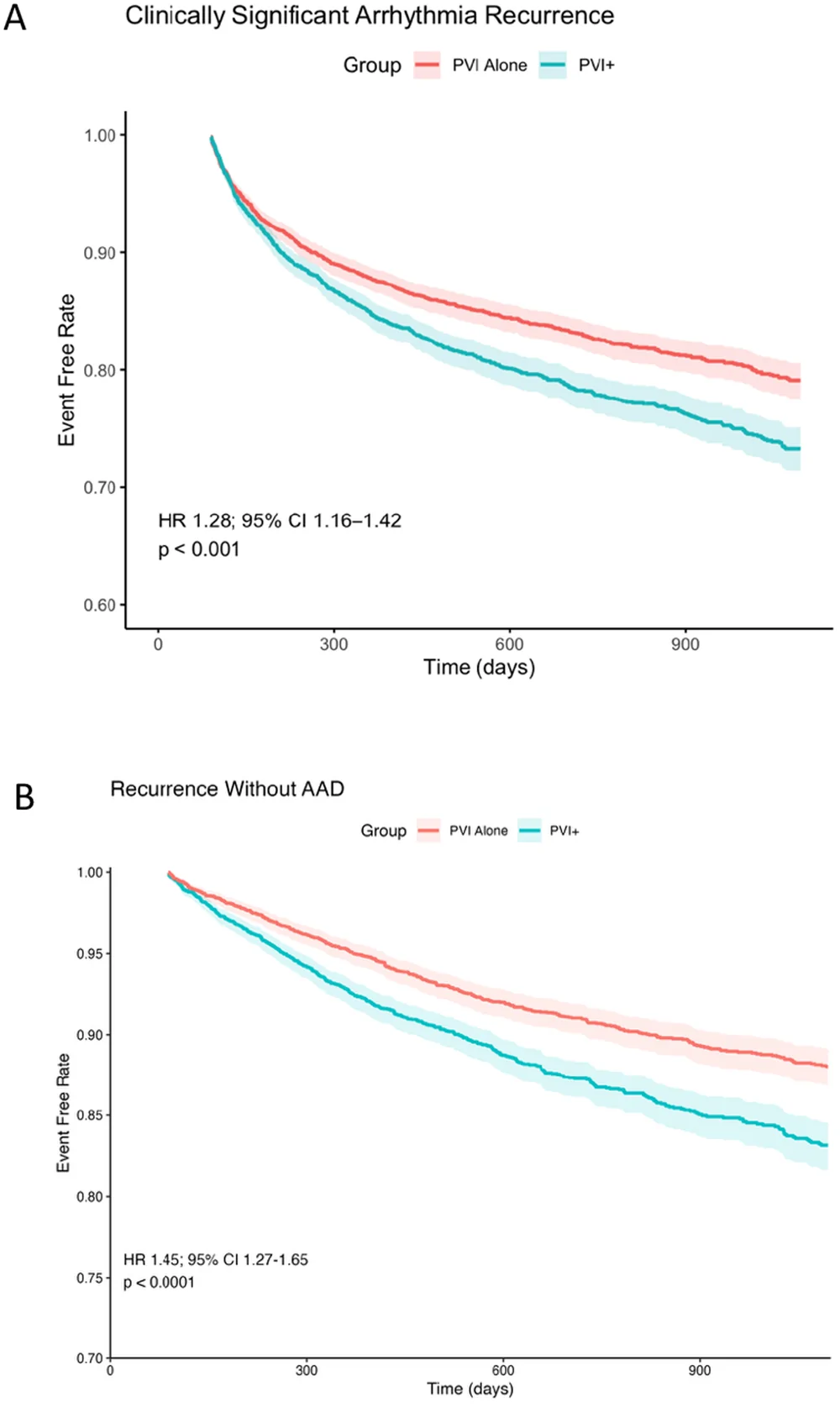
Kaplan–Meier analysis of clinically significant atrial arrhythmia recurrence after pulmonary vein isolation alone vs. PVI plus additional ablation in paroxysmal atrial fibrillation. (A) Recurrence defined as repeat ablation, cardioversion, or antiarrhythmic drug (AAD) use. (B) Recurrence defined as repeat ablation or cardioversion only, excluding AAD use.

### 3.3 Secondary Outcomes

#### 3.3.1 Repeat Ablation

Repeat ablation occurred in 219 patients (4.4%) in the PVI+ group and 136 patients (2.7%) in the PVI‐only group. Kaplan–Meier analysis showed a significantly higher cumulative incidence of redo ablation in the PVI+ group (log‐rank *p* < 0.001). On Cox regression, PVI+ was associated with a nearly twofold increased risk of repeat ablation (HR 1.99; 95% CI 1.60–2.46) (Figure 2).

**Figure 2:**
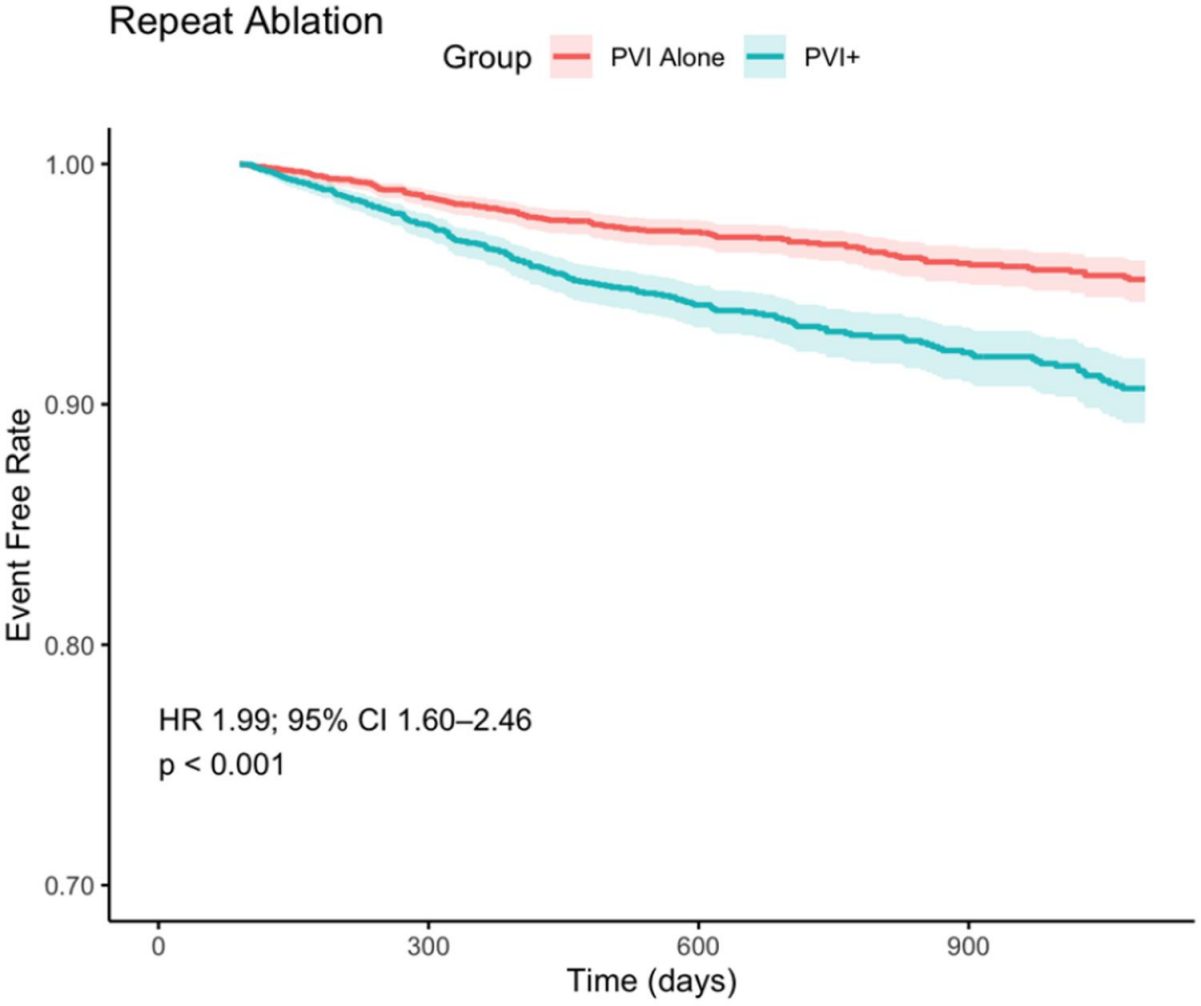
Kaplan–Meier Analysis of repeat ablation procedures after pulmonary vein isolation alone vs. PVI plus additional ablation in paroxysmal atrial fibrillation.

#### 3.3.2 Electrical Cardioversion

Electrical cardioversion occurred in 222 patients (4.4%) in the PVI+ group and 192 patients (3.8%) in the PVI‐only group. Time‐to‐event analysis demonstrated a significantly higher cumulative incidence in the PVI+ cohort (log‐rank *p* = 0.001). Cox proportional hazards modeling showed a 37% higher risk of cardioversion with PVI+ (HR 1.37; 95% CI 1.13–1.66) (Figure 3).

**Figure 3:**
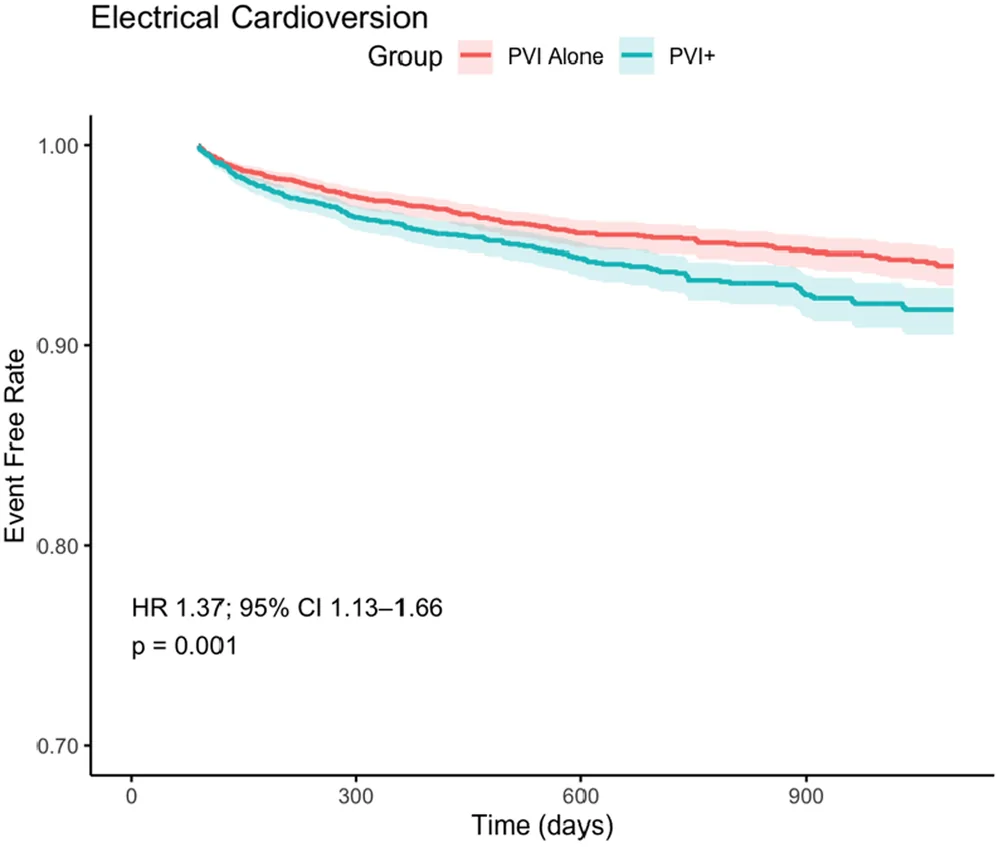
Kaplan–Meier analysis of electrical cardioversion after pulmonary vein isolation alone vs. PVI plus additional ablation in paroxysmal atrial fibrillation.

#### 3.3.3 Antiarrhythmic Drug Usage

Among patients without antiarrhythmic drug usage prior to the time window, post‐blanking usage occurred in 98 patients (3.7%) in the PVI+ group and 88 patients (3.2%) in the PVI‐only group. Kaplan–Meier analysis demonstrated a significantly higher cumulative incidence in the PVI+ group (log‐rank *p* = 0.020), with Cox regression showing a 40% increased risk of antiarrhythmic drug usage (HR 1.41; 95% CI 1.05–1.87) (Figure 4).

**Figure 4:**
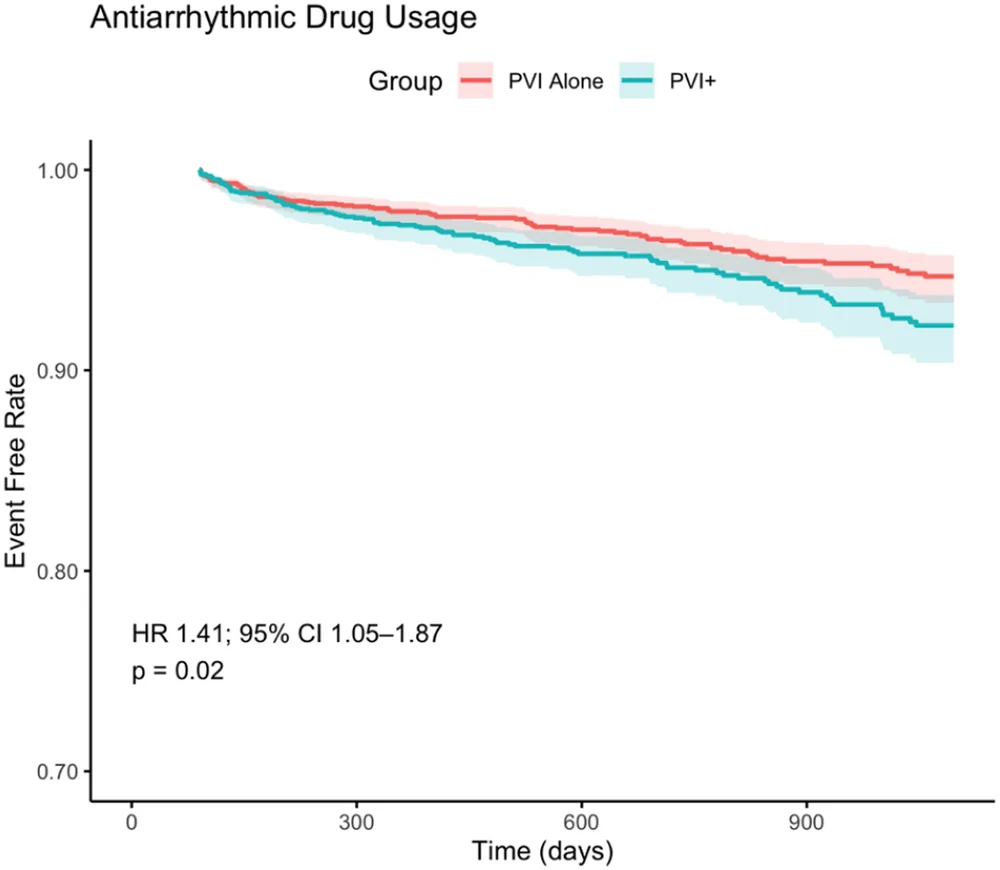
Kaplan–Meier analysis of antiarrhythmic drug usage after pulmonary vein isolation alone vs. PVI plus additional ablation in paroxysmal atrial fibrillation.

#### 3.3.4 Progression to Persistent Atrial Fibrillation

Progression to persistent atrial fibrillation occurred in 725 patients (14.4%) in the PVI+ group and 609 patients (12.1%) in the PVI‐only group. Kaplan–Meier analysis showed a significantly higher cumulative incidence in the PVI+ cohort (log‐rank *p* < 0.001). In Cox regression, PVI+ was associated with a 37% increased risk of progression (HR 1.37; 95% CI 1.23–1.53) (Figure 5).

**Figure 5:**
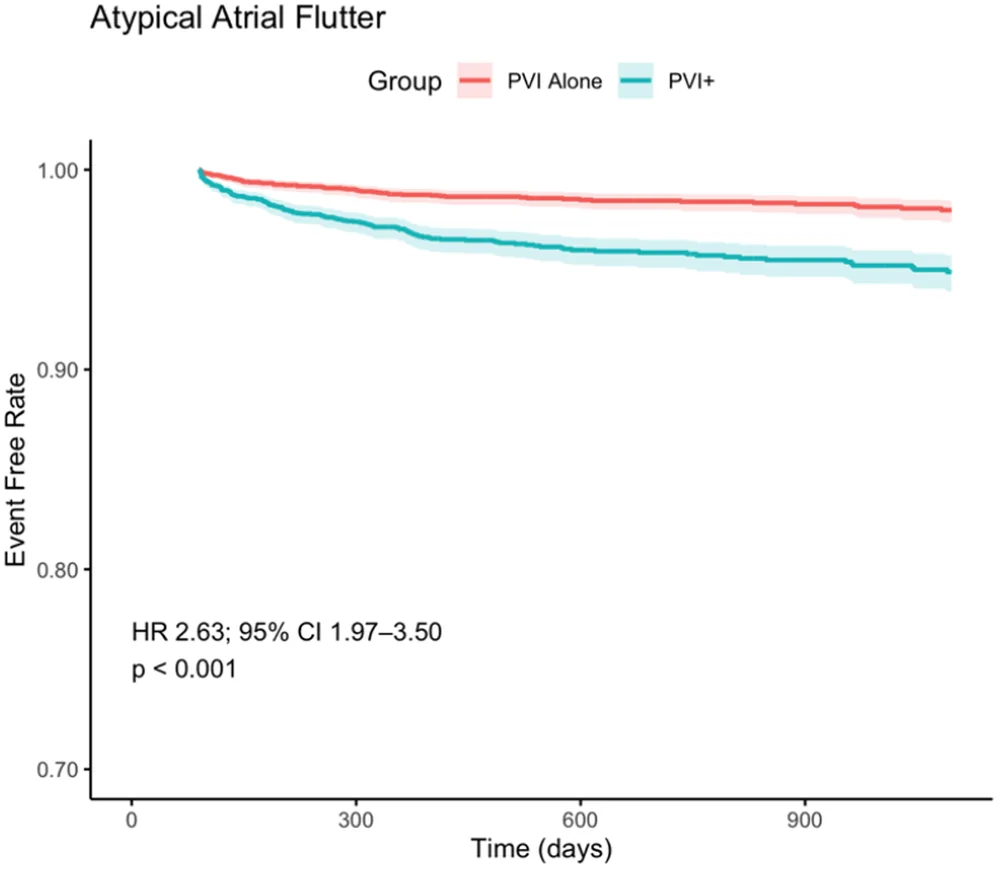
Kaplan–Meier analysis of atypical atrial flutter after pulmonary vein isolation alone vs. PVI plus additional ablation in paroxysmal atrial fibrillation.

#### 3.3.5 Atypical Atrial Flutter

Atypical atrial flutter occurred in 151 patients (3.0%) in the PVI+ group compared with 67 patients (1.3%) in the PVI‐only group. Kaplan–Meier analysis demonstrated a significantly higher cumulative incidence in the PVI+ group (log‐rank *p* < 0.001). On Cox proportional hazards analysis, PVI+ was associated with a more than twofold increased risk of atypical flutter (HR 2.63; 95% CI 1.97–3.50) (Figure 6).

**Figure 6:**
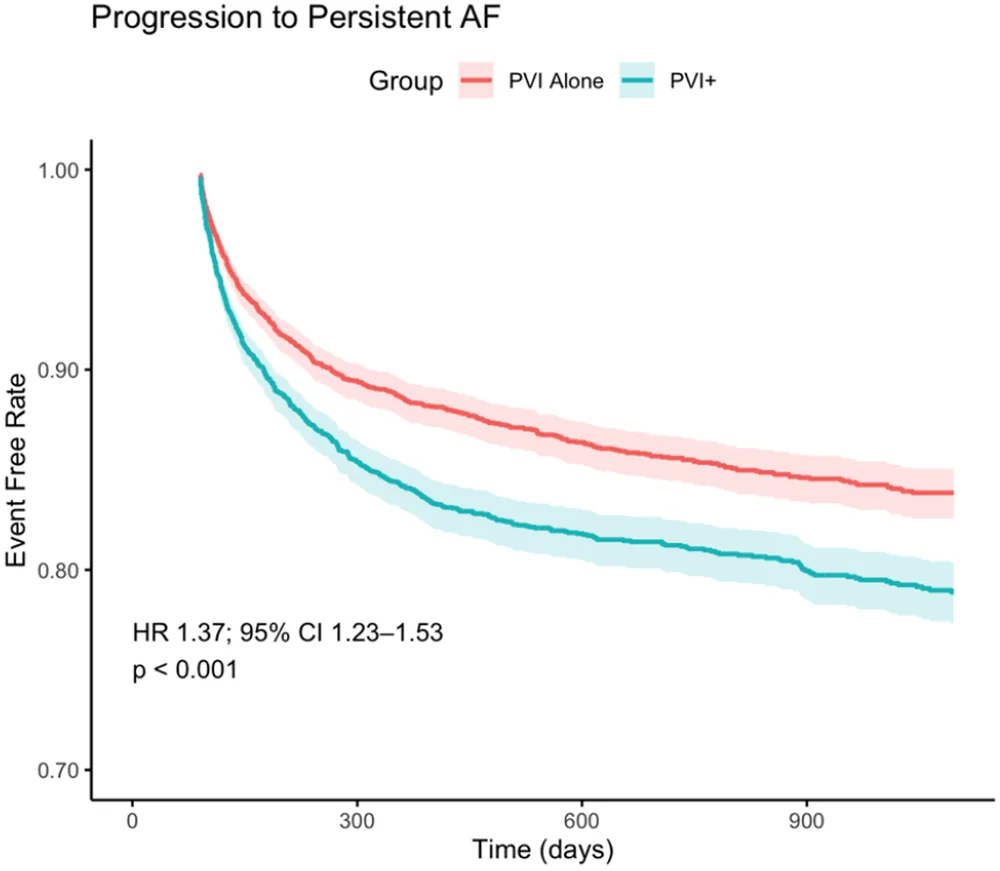
Kaplan–Meier analysis of progression to persistent atrial fibrillation after pulmonary vein isolation alone vs. PVI plus additional ablation in paroxysmal atrial fibrillation.

## 4 Discussion

### 4.1 Main Findings

In this large, real‐world analysis, we noted that more than a quarter of PAF patients underwent PVI+ additional ablation (PVI+), a finding that is striking given the absence of guideline support or consistent randomized evidence favoring this approach. This strategy was found in a propensity‐matched analysis to be associated with a significant increase in AF recurrence, repeat ablation, AAD use, or cardioversion when compared to standard of care PVI only strategy. Additionally, PVI+ was associated with more frequent progression from paroxysmal AF to persistent AF and a higher incidence of atypical atrial flutter following ablation. These findings reinforce contemporary guidelines that recommend PVI as the primary treatment strategy for PAF.

### 4.2 Mechanistic and Disease‐Stage Rationale for PVI in Paroxysmal AF

The pulmonary vein–left atrial junction plays a central role in atrial fibrillation pathophysiology due to its propensity for abnormal automaticity and triggered activity, making it a dominant source of ectopic firing in paroxysmal AF [6, 7, 8]. Contemporary clinical practice guidelines consistently endorse PVI as the first‐line ablation strategy for patients with PAF, reflecting a robust body of mechanistic, randomized, and clinical outcomes data demonstrating that durable rhythm control can be achieved by targeting pulmonary vein triggers alone [4]. The 2023 ACC/AHA/ACCP/HRS guidelines explicitly acknowledge that multiple ablation targets beyond the pulmonary veins, including the posterior left atrial wall, left atrial appendage, ligament of Marshall, and atrial scar, have been evaluated in randomized control trials and observational studies with no adjunctive strategy demonstrating consistent benefit across patient populations. Accordingly, there is no recommendation for routine ablation beyond traditional PVI for PAF.

Randomized controlled trials have provided strong clinical support for this mechanistic framework. RAAFT‐2, MANTRA‐PAF and FIRE AND ICE consistently demonstrated higher rates of freedom from recurrent atrial arrhythmias with catheter ablation strategies centered on pulmonary vein isolation compared with antiarrhythmic drug therapy, establishing PVI as the cornerstone of rhythm control in early disease [9, 10, 11]. Three subsequent landmark trials, EARLY‐AF, STOP AF First, and Cryo‐FIRST, extended this evidence to treatment‐naïve PAF patients, each demonstrating that first‐line cryoballoon PVI significantly reduced atrial arrhythmia recurrence compared with antiarrhythmic drugs, with freedom from arrhythmia ranging from 57% to 82% in ablation arms vs. 32% to 68% in drug arms [3, 12, 13]. Three‐year follow‐up of EARLY‐AF further showed that initial cryoablation reduced progression to persistent AF (1.9% vs. 7.4%; HR 0.25) and hospitalizations compared with drug therapy [14]. Collectively, these trials reinforce that PVI alone, without additional lesion sets, is sufficient to achieve durable rhythm control in a large proportion of patients with early PAF.

Disease stage strongly influences ablation effectiveness. Paroxysmal atrial fibrillation is predominantly trigger‐driven, with limited structural remodeling, making it highly amenable to pulmonary vein isolation alone. In contrast, persistent AF is associated with more advanced atrial remodeling and substrate‐dependent mechanisms that may reduce the efficacy of trigger elimination alone. Prior studies show 60%–80% freedom from atrial fibrillation at 1 year in paroxysmal atrial fibrillation compared with 40%–60% in persistent atrial fibrillation, highlighting both the efficacy of early intervention and diminishing returns as disease progresses [15, 16]. While most ectopy in paroxysmal atrial fibrillation arises from the pulmonary veins, a minority of patients harbor extra–pulmonary vein triggers, and identifying this subgroup remains an active area of investigation (Central Illustration 1).

**Central illustration 1:**
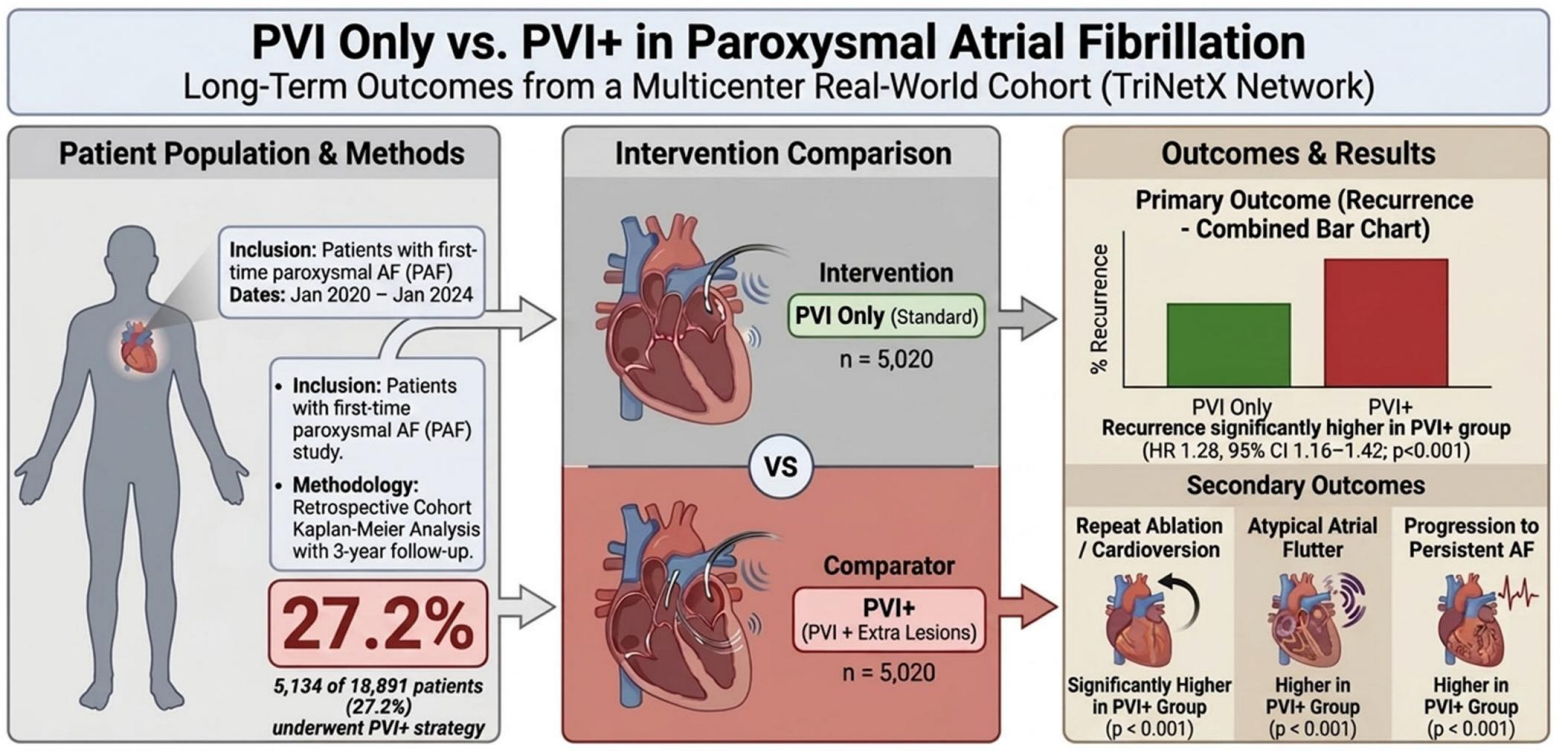
Clinical outcomes of pulmonary vein isolation alone vs. additional lesion sets in paroxysmal atrial fibrillation.

### 4.3 The Prevalence of Extra‐PV Ablation in PAF: A Practice‐Evidence Gap

One of the most striking findings of this study is that more than a quarter of patients with paroxysmal AF underwent ablation beyond PVI. This rate is remarkable and highlights a profound gap between evidence‐based guideline recommendations and real‐world clinical practice. Several randomized controlled trials have directly evaluated adjunctive ablation strategies beyond PVI specifically in paroxysmal AF populations, and the preponderance of evidence does not support their use. The VOLCANO trial randomized 62 PAF patients with left atrial low‐voltage areas (LVAs) to PVI plus LVA ablation versus PVI alone. At 1 year, LVA ablation conferred no benefit in AF‐recurrence‐free survival (57% vs. 53%, *p* = 0.67), despite longer procedural and fluoroscopic times in the LVA ablation group [17]. Extended follow‐up at a median of 25 months confirmed no impact on long‐term rhythm outcomes (57% vs. 59% recurrence, *p* = 0.83) and revealed that LVA ablation significantly increased the incidence of iatrogenic atrial tachycardias (71% vs. 32%, *p* < 0.0001), demonstrating that additional lesion sets in PAF may be not only ineffective but proarrhythmic [18].

The AWARE trial randomized 398 PAF patients to standard single wide‐area circumferential ablation (WACA) with adenosine testing versus augmented double WACA. At 1 year, there was no difference in atrial arrhythmia recurrence between groups (26.7% vs. 24.6%; relative risk 0.92, 95% CI 0.66–1.29, *p* = 0.64), with no benefit observed across any prespecified subgroup. The authors concluded that there may be a ceiling effect to single‐procedure ablation success in PAF, beyond which additional empirical tissue destruction does not improve outcomes [19].

Our findings in the US echo similar findings noted in European registries. The ESC‐EORP EHRA AF Ablation Long‐Term Registry found that approximately 19% of PAF patients in Europe underwent PVI+ strategies, with no improvement in arrhythmia‐free survival compared to PVI alone and higher rates of AF‐related hospital readmissions in the PVI+ group [20]. Our data directly challenge the assumption that more extensive ablation equates to better outcomes and demonstrate that in PAF, extra‐PV ablation is associated with worse outcomes, including higher rates of AF recurrence, progression to persistent AF, and iatrogenic atypical atrial flutter.

### 4.4 Safety and Proarrhythmic Concerns of Adjunctive Ablation

In addition to the lack of proven efficacy, adjunctive ablation carries important safety and proarrhythmic considerations. Additional atrial lesions may increase the risk of organized atrial tachyarrhythmias by creating regions of slow conduction or incomplete block, facilitating macroreentrant circuits [21]. Superior vena cava isolation and extensive substrate modification have also been associated with increased risks of phrenic nerve injury, sinus node dysfunction, and iatrogenic atrial tachycardia [22]. The extended follow‐up of the VOLCANO trial provides direct evidence of this proarrhythmic risk, with LVA ablation significantly increasing the incidence of atrial tachycardia in PAF patients [18]. Our findings of significantly higher rates of atypical atrial flutter and progression to persistent atrial fibrillation in the PVI+ group provide large‐scale, real‐world clinical evidence supporting these mechanistic concerns and suggest that additional lesion sets in early, trigger‐driven disease may not confer benefit and could carry additional proarrhythmic risk, though residual confounding by indication cannot be excluded.

### 4.5 Limitations

This study is limited by its retrospective design and reliance on ICD/CPT coding. Specifically, reliance on CPT coding for ablation in this context cannot distinguish more granularly between PVI+ adjunctive lesion sets or other procedure related factors (e.g., Lesion duration, durability, or ablation energy modality). Additionally, the reliance on a single PAF code does not recognize the heterogeneity in AF burden of patients with paroxysmal disease. Furthermore, it is plausible that some patients with only PAF coding had indeed persistent AF that was never codified in the medical record. Residual confounding and confounding by indication should be considered as operators may preferentially perform PVI+ in patients with more complex atrial substrate not fully captured in the demographics measured (left atrial size, fibrosis, reason to suspect non‐pulmonary vein triggers, etc). PAF burden prior to ablation was similarly unavailable and may have influenced both the decision to perform PVI+ and the observed outcomes. Asymptomatic AF recurrence, which is not captured by claims‐based outcome definitions, may also have been underascertained in both cohorts, and heterogeneity across the 109 participating HCOs in rhythm‐monitoring intensity, follow‐up frequency, and coding practices for adjunctive ablation may further limit comparability.

### 4.6 Future Directions

The advent of pulsed field ablation (PFA) with a favorable collateral injury profile compared with thermal energy, has accelerated adoption and broadened operator comfort with additional left atrial lesion sets. The concern has been raised about potential harms of this perceived “comfort” in ablating more extensively with this new technology. Expanding lesion burden may worsen AF patient outcomes via multiple pathways including proarrhythmia in patients with incomplete lines of block, and accelerated atrial myopathy from atrial injury/scar. Furthermore, increasing ablation burden can predispose to ablation related complications including atrioesophageal fistula and phrenic nerve palsy with radiofrequency ablation, or hemolysis, stroke, and ischemic complications in PFA patients.

## 5 Conclusion

In this large, real‐world analysis, adjunctive ablation beyond PVI during first‐time catheter ablation for PAF was surprisingly common and occurred in more than a quarter of patients. This strategy was associated with higher rates of arrhythmia recurrence, repeat ablation, cardioversion, AAD use, progression to persistent AF and atypical atrial flutters compared with PVI alone. Our findings reinforce the current guideline recommendations supporting PVI as the primary, evidence‐based ablation strategy for patients with PAF (Tables 1 and 2).

## Funding

The authors have nothing to report.

## Ethics Statement

This study used de‐identified data from the TriNetX Research Network. As investigators had no access to identifiable patient information, the study was exempt from institutional review board approval and the requirement for informed consent.

## Conflicts of Interest

N.F.M. reports having received consulting fees from Biosense Webster, Boston Scientific and AtriCure. N.F.M. also reports being a speaker for Abbott, Biosense Webster, AtriCure and Sanofi. N.F.M. also reports receiving research support Abbott, Medtronic, Biosense Webster, Siemens, GE, Boston Scientific, Sanofi, and Samsung. N.F.M. also reports having a family member as the CEO of Cardiac Designs. N.F.M. also reports being the founder of Marrek, being named in a patent issued for MRI fibrosis imaging and being a previous shareholder of Cardiac Designs. A.F.P. reports having served on an advisory board for Novartis (completed). All other co‐authors have no relevant conflict of interest.

## Supporting information


**Table S1:** Baseline Characteristics Before Propensity Score Matching.
