# Supplementary material for: Pulmonary Vein Isolation Alone Versus Adjunctive Ablation for Paroxysmal Atrial Fibrillation
Source: J Cardiovasc Electrophysiol. 2026 Oct 6;37(11):2740–50. doi: 10.1111/jce.70519 (PMC13768794; doi:10.1111/jce.70519)
Supplement: Supplementary file 1 [file JCE-37-2740-s001.docx]

**Table S1. Baseline Characteristics Before Propensity Score Matching**

| **Variable** | **PVI+ (n=5,268)** | **PVI-Only (n=14,046)** | **p-value** | **SMD** |
| --- | --- | --- | --- | --- |
| Age, mean ± SD | 67.4 ± 10.5 | 64.5 ± 11.1 | <0.001 | 0.270 |
| Female sex, % | 43.9% | 41.0% | <0.001 | 0.060 |
| White race, % | 87.4% | 87.3% | 0.789 | 0.004 |
| Black race, % | 5.4% | 4.7% | 0.033 | 0.034 |
| Hypertension, % | 75.5% | 64.8% | <0.001 | 0.235 |
| Diabetes, % | 22.6% | 19.6% | <0.001 | 0.073 |
| COPD, % | 10.3% | 7.5% | <0.001 | 0.097 |
| Heart failure, % | 28.2% | 17.7% | <0.001 | 0.252 |
| Ischemic heart disease, % | 45.6% | 35.7% | <0.001 | 0.201 |
| OSA, % | 34.1% | 30.3% | <0.001 | 0.081 |
| BMI, mean ± SD | 30.4 ± 6.4 | 30.5 ± 6.4 | 0.438 | 0.015 |
| Antiarrhythmic use, % | 80.6% | 74.0% | <0.001 | 0.158 |
| Anticoagulation, % | 89.6% | 87.0% | <0.001 | 0.080 |

*Cohort counts reflect TriNetX's live federated query at the time PSM was performed; a minor variation from the originally defined cohort sizes (5,134 / 13,757) is expected given the platform's real-time re-querying between cohort definition and analysis steps. This does not affect the matched analysis, where both cohorts are identical in size (n=5,020) and match the results reported in the manuscript.*
